# Draft genome sequence of a nontypeable *Haemophilus influenzae* strain used in the study of human respiratory infection

**DOI:** 10.1186/s13104-021-05528-5

**Published:** 2021-04-01

**Authors:** Rajendra KC, Ronan F. O’Toole

**Affiliations:** 1 Menzies Institute for Medical Research, University of Tasmania, Tasmania, 7005 Australia; 2 Department of Pharmacy and Biomedical Sciences, School of Molecular Sciences, College of Science, Health and Engineering, La Trobe University, Victoria, 3690 Australia

**Keywords:** Whole genome sequence, Nontypeable *Haemophilus influenzae*, Chronic obstructive pulmonary disease

## Abstract

**Objectives:**

Nontypeable *Haemophilus influenzae* (NTHi) is an important human respiratory bacterium that can cause a range of diseases including sinusitis, otitis media, conjunctivitis, pneumonia as well as acute exacerbations of chronic obstructive pulmonary disease (COPD). A number of studies have used NTHi clinical isolate RHH-3 as a laboratory strain for experimentation examining the effect of cigarette smoke and more recently, biomass smoke, on the susceptibility and response of cells lining the respiratory tract to infection. Therefore, definition of the genome content of RHH-3 is required to fully elucidate human-NTHi interactions associated with initial infection and subsequent development of respiratory disease.

**Data description:**

Here, we present the draft genome sequence of NTHi RHH-3 collected from the sputum of a patient at the Royal Hobart Hospital, Tasmania, Australia. The assembled genome size was 1,839,376 bp consisting of 61 contigs (> 500 bp), with a G+C content of 38.1%. This draft genome data can be accessed at DDBJ/ENA/GenBank under the accession number JADPRR000000000.

## Objective

Nontypeable *Haemophilus influenzae* (NTHi) strains are common commensal inhabitants of the human nasopharynx. However, they can spread to the sinuses or middle ear via the eustachian tube causing sinusitis and otitis media, respectively, and can also migrate to the eyes causing conjunctivitis [1, 2]. Moreover, they can penetrate into the nasopharyngeal mucosa, or descend to the lower regions of the respiratory tract, resulting in invasive infections that include septicaemia and meningitis, or non-invasive infections such as pneumonia and exacerbations of COPD [3–5]. Environmental factors, such as exposure to tobacco or biomass smoke have been found to increase susceptibility to infection by respiratory bacteria such as NTHi [6–8]. NTHi strain RHH-3 has been used in mechanistic studies investigating how tobacco and biomass smoke exposure increases the risk of airway infection [9]. The draft assembled genome sequence of NTHi RHH-3 presented here will enable more in-depth studies to be conducted on specific genes that promote NTHi survival and propagation in the COPD lung or that contribute to inflammation that results in tissue impairment and disease. This will provide further insights into the role of NTHi infection in the pathogenesis of COPD. It would also be interesting to investigate in future work whether exposure of lung tissue to smoke predisposes an individual to colonization by a subset of NTHi strains, given a recent finding from pan-genome-wide association analysis that certain NTHi accessory genes are significantly associated with COPD [10].

## Data description

NTHi strain RHH-3 was isolated from the sputum of a patient presenting with lower respiratory tract infection at the Royal Hobart Hospital, Australia [9, 11]. The sputum sample was homogenized and cultured on chocolate blood agar plates at 35 °C in a CO_2_ atmosphere as previously described [12]. Isolated Gram-negative rod colonies, with small and translucent colony morphologies suggestive of *Haemophilus* species, were identified as NTHi through the use of matrix-assisted laser desorption ionization-time of flight mass spectrometry (MALDI-TOF MS, Bruker Daltonics GmbH, Leipzig, Germany). The isolate was then grown overnight on chocolate agar, incubated at 35 °C with 5% CO_2_. A single colony from a chocolate agar plate was suspended in 200 µL PBS and then genomic DNA was extracted using the DNeasy Blood and Tissue Kit (Catalog number 69504; Qiagen, USA). Genomic DNA was further purified using the High Pure PCR Template Preparation Kit (Catalog number 11796828001; Roche, Germany). DNA library preparation was performed using a Nextera XT DNA library preparation kit (Catalog number FC-131-1024; Illumina, USA) as described previously [10, 13, 14]. Sequencing was performed using a MiSeq Reagent Kit v2 (300-cycles) (Catalog number MS-102-2002) with 150-bp paired-end sequencing as previously described [12]. In total, 933,328 paired-end reads were generated, representing an average read depth of 73.13-fold (Table 1). Reads were trimmed of adapters using Trimmomatic [15] and de novo assembly of reads was performed with SPAdes v3.12.0 [16]. All parameters were set to default except for the size of k-mers which were manually chosen as 21, 33, 43, 53, 63, 75. This resulted in the generation of a 1,839,376 bp draft genome consisting of 61 contigs (≥ 500 bp) that covered 82.74% of the *H. influenzae* 86-028NP genome, a well-studied NTHi isolate (Table 2) [17]. The N50 contig was 52,548 bp, and the overall GC content was 38.1% (Table 2). The genome assembly quality, including completeness with respect to the 86-028NP genome, was determined using the QUAST quality assessment tool [18]. In addition, the RHH-3 genome was estimated as 99.77% complete with 0% contamination by CheckM [19].

**Table 1:** Overview of data sets

Label	Name of data file/data set	File types (file extension)	Data repository and identifier (DOI or accession number)
Draft genome assembly	NTHi strain RHH-3, whole genome sequence	FASTA	Genbank (https://www.ncbi.nlm.nih.gov/nuccore/JADPRR000000000.1/) [26]
16S ribosomal RNA gene, partial sequence	NTHi strain RHH3, 16S rRNA gene sequence	FASTA	Genbank (https://www.ncbi.nlm.nih.gov/nuccore/MW255938.1/) [27]

**Table 2:** Genome assembly/annotation statistics

Total sequence length	1,839,376
Number of contigs	138
Contig N50	52,548
Contig L50	12
GC content	38.1%
Coverage of *H. influenzae* 86-028NP genome	82.74%
Total number of genes	1959
Number of protein coding sequences	1907
Number of RNA genes	52

The identity of strain RHH-3 was confirmed by its 16S ribosomal RNA gene sequence (Table 1) (16S rRNA gene sequence, 1543 bp, BLAST identity of 99.48% to *H. influenzae* strain NCTC11931 accession: LS483392.1). The draft sequence of RHH-3 was submitted to the *H. influenzae* multi-locus sequence typing (MLST) website (https://pubmlst.org/hinfluenzae/) for the purposes of generating an in silico MLST profile [20]. The allelic profile of seven housekeeping genes used in the *H. influenzae* MLST was well-defined in RHH-3 *i.e., adk*_98, *atpG*_2, *frdB*_70, *fucK*_15, *mdh*_310, *pgi*_158, and *recA*_4 however, the combination of these alleles was novel as an MLST sequence type corresponding to this allele profile was not available in the *H. influenzae* MLST database (https://pubmlst.org/organisms/haemophilus-influenzae). Based on its unique allele profile, RHH-3 has been assigned an MLST sequence type ST-2380. It is common for NTHi strains to have diverse MLST types due to a relatively high rate of recombination across the genome [21, 22]. Gene prediction and annotation was performed using the Rapid Annotation System Technology (RAST) server [23–25], which identified a total of 1,959 genes consisting of 1,907 protein coding sequences, and 5 rRNA and 47 tRNA genes (Table 2). Default parameters were used for all software unless otherwise specified.

## Limitations

Comparative analyses were not performed and further investigations are needed to determine the relatedness of RHH-3 to a diverse range of other NTHi isolates.

## Data Availability

The data described in this Data note can be freely and openly accessed at DDBJ/ENA/GenBank. Accession numbers-https://www.ncbi.nlm.nih.gov/nuccore/JADPRR000000000.1/ (whole genome sequence) and https://www.ncbi.nlm.nih.gov/nuccore/MW255938.1/ (16S ribosomal RNA gene sequence). The associated BioProject, SRA, and BioSample accession numbers are PRJNA678621, SRR13065832 and SAMN16808213, respectively. Please see Table 1 and references [26, 27] for details and links to the data.

## Abbreviations

NTHi: Nontypeable *Haemophilus influenzae*
COPD: Chronic obstructive pulmonary disease
MLST: Multilocus sequence typing
rRNA: Ribosomal RNA
